# Clinical analysis of complete uterine rupture during pregnancy

**DOI:** 10.1186/s12884-024-06394-2

**Published:** 2024-04-08

**Authors:** Jing Xie, Xuefang Lu, Miao Liu

**Affiliations:** 1 The Chenzhou No.1 People’s Hospital, Chenzhou, 423000 China; 2 The First Affiliated Hospital of Xiangnan University, Chenzhou, 423000 China; 3 Department of Radiology, Renmin Hospital of Wuhan University, No. 238 Jiefang Road, Wuchang District, Wuhan, 430060 China

**Keywords:** Complete uterine rupture, hysterectomy, pregnancy

## Abstract

**Background:**

Uterine rupture in pregnant women can lead to serious adverse outcomes. This study aimed to explore the clinical characteristics, treatment, and prognosis of patients with complete uterine rupture.

**Methods:**

Data from 33 cases of surgically confirmed complete uterine rupture at Chenzhou No.1 People’s Hospital between January 2015 and December 2022 were analyzed retrospectively.

**Results:**

In total, 31,555 pregnant women delivered in our hospital during the study period. Of these, approximately 1‰ (*n* = 33) had complete uterine rupture. The average gestational age at complete uterine rupture was 31^+4^ weeks (13^+1^–40^+3^ weeks), and the average bleeding volume was 1896.97 ml (200–6000 ml). Twenty-six patients (78.79%) had undergone more than two deliveries. Twenty-five women (75.76%) experienced uterine rupture after a cesarean section, two (6.06%) after fallopian tube surgery, one (3.03%) after laparoscopic cervical cerclage, and one (3.03%) after wedge resection of the uterine horn, and Fifteen women (45.45%) presented with uterine rupture at the original cesarean section incision scar. Thirteen patients (39.39%) were transferred to our hospital after their initial diagnosis. Seven patients (21.21%) had no obvious symptoms, and only four patients (12.12%) had typical persistent lower abdominal pain. There were 13 cases (39.39%, including eight cases ≥ 28 weeks old) of fetal death in utero and two cases (6.06%, both full term) of severe neonatal asphyxia. The rates of postpartum hemorrhage, blood transfusion, hysterectomy were 66.67%, 63.64%, and 21.21%. Maternal death occurred in one case (3.03%).

**Conclusions:**

The site of the uterine rupture was random, and was often located at the weakest point of the uterus. There is no effective means for detecting or predicting the weakest point of the uterus. Rapid recognition is key to the treatment of uterine rupture.

## Background

Uterine rupture(UR) is a serious complication that directly jeopardizes the life of the mother and the fetus [1]. It refers to the rupture of the uterine body or the lower uterine segment in late pregnancy or during labor [2], requiring a cesarean section to terminate the pregnancy as soon as the diagnosis is confirmed. The most common risk factors of UR are a history of previous cesarean section (CS), myomectomy, multiparity, malpresentation, breech extraction, and instrumental deliveries [3].

The incidence of uterine rupture in China has recently been reported to range from 0.1% to 0.55% [4]; although this incidence rate is low, UR is highly likely to lead to serious adverse outcomes.

Currently, there are no effective means for detecting or predicting the weakest points of the uterus. Therefore, in this study, we aimed to provide reference information and practical experience for the early recognition, management and emergency treatment of uterine rupture.

## Methods

The Department of Obstetrics and Gynaecology of the First people’s Hospital of Chenzhou is a critical care center wherein treatment, consultation, referral, and technical guidance are proviede to pregnant women in Southern Hunan and the city with acute and critical illnesses. This was a retrospective study aimed at exploring the clinical characteristics, treatment, and prognosis of patients with complete uterine rupture between January 2015 and December 2022. The data (complete clinical data, medical history, and surgical records) of all patients with complete uterine rupture admitted to our hospital were retrospectively analyzed.

### Diagnostic criteria

Complete uterine rupture was defined as rupture of the entire wall of the uterine myometrium, with the uterine cavity communicating with the abdominal cavity during late pregnancy or labor [1].

Postpartum hemorrhage (PPH) was defined as bleeding of ≥ 500 ml for vaginal delivery and ≥ 1000 ml for cesarean delivery within 24 h after delivery of the fetus [1].

### Research methods

Basic maternal information (age, pregnancies, number of deliveries), previous pregnancy and surgery-related indicators (risk factor, causes and clinical manifestations, comorbidities, distance between periconceptional caesarean section scar and vesicovaginal fold), situation at the time of uterine rupture (gestational age, interval between the current pregnancy and previous cesarean section delivery, rupture site and length, bleeding volume and number of required blood transfusions, minimum hemoglobin level), mode of the current delivery (induced delivery, transvaginal delivery, or cesarean section), and outcomes of the mother and child (postpartum hemorrhage, hysterectomy, maternal death, perinatal deaths, severe neonatal asphyxia (Apgar scores are recorded at 1, 5, and 10 min after birth, with a score below or equal to 3 indicating severe asphxia) were cllected from the patients’ medical records. This study meticulously adheres to the Strengthening the Reporting of Observational Studies in Epidemiology (STROBE) Statement guidelines.

## Results

### General information

This was a retrospective study aimed at exploring the clinical characteristics, treatment, and prognosis of all patients with complete uterine rupture between January 2015 and December 2022. The data (complete clinical data, medical history, and surgical records) of all patients with uterine rupture admitted to our hospital were retrospectively analyzed. Thirty-three patients with surgically confirmed complete uterine rupture were included into the study.

### Incidence of uterine rupture in our hospital

The total number of pregnant women who delivered in our hospital during the study was 31,555, with 33 cases of complete uterine rupture, accounting for approximately 0.1%(Table 1). The average gestational age at complete uterine rupture was 31^+4^ weeks (13^+1^–40^+3^ weeks), and the average bleeding volume was 1896.97 ml (200–6000 ml) (Table 2).

**Table 1:** Deliveries in our hospital from January 2015 to December 2022

Year	Number of births	Number of uterine ruptures	Rate (%)
2015	3766	8	0.21
2016	4375	3	0.07
2017	4461	2	0.05
2018	4294	2	0.05
2019	4315	4	0.09
2020	3581	6	0.17
2021	3368	4	0.12
2022	3395	4	0.12
total	31,555	33	0.10

**Table 2:** Data of 33 cases of complete uterine rupture

Serial number	Age (y)	Gestation week (w)	No. of pregnancies	CS-VVF(mm)	Risk factor	Time since previous CS (month)	Location and size of the breach	Bleeding volume (ml) and blood transfusions	Causes and clinical manifestations	Comorbidities and outcomes^b^	Minimum hemoglobin (g/L)
1	27	33^+3^	2	25.8	CS1, mental retardation	13	Original CS scar, 10 cm	intraperitoneal 900, hemorrhage 100	Bloating 8 + h	stillbirth	98
2	35	29^+1^	6	22.1	CS1, AA4	38	Multiple large ruptures of the uterus (original CS scar, lower back wall, left side wall)	intraperitoneal 1600, hemorrhage 500 erythrocyte 10 U	Coma total hysterectomy	Stillbirth, traffic accidents	50
3	31	38	7	27.5	VB4, CS2	36	Original CS scar right side, 5 cm	Intraperitoneal 3000, hemorrhage 3000 erythrocyte 14 U, plasma 600 ml	Vaginal hemorrhage after delivery 4 h subtotal hysterectomy	10′ 3500 g PI	57
4	32	36^+1^	2	38.5	CS1 (Superimposed Preeclampsia)	75	Original CS scar, 2 cm	Intraperitoneal 1500, hemorrhage 200 erythrocyte 6 U, plasma 600 ml	Persistent abdominal pain after spontaneous delivery of a stillborn baby	stillbirth CHWSP	61
5	26	38^+5^	2	26.9	CS1	34	Original CS scar, 2 cm	intraperitoneal 100, hemorrhage 600	Painless vaginal bleeding 1 day	8’-10′ 3300 g CPP + PI	102
6	32	26^+3^	3	22.7	CS1 (twins, PPH), AA1, 2013 Laparoscopy for EMT	13	Original CS scar, 10 cm	intraperitoneal 2000, hemorrhage 2000 erythrocyte 14 U, plasma 1000 ml	Bloating 8 + h total hysterectomy	stillbirth	70
7	45	29^+3^	6	38.5	VB4, CS1, Double uterus (single cervix, double uterine cavity)	58	Right uterine corpus, 15 cm, (up to the uterine fundus and down to the endocervix)	intraperitoneal 2000, hemorrhage 200 erythrocyte 8 U, plasma 600 ml	abdominal pain 7 + h Removal of the body of the uterus on the sick side	stillbirth CH	69
8	30	29	3	-	VB1 (CPP, PPH), AA1, curettage 1 (retained placenta)	—	Uterine fundus, 10 cm	intraperitoneal 200, hemorrhage 3000 erythrocyte 8 U, plasma 400 ml	Persistent epigastric pain 1 + day total hysterectomy	9’-10′ 1400 g PI	43
9	27	33	5	38.9	CS1, AA3	37	Original CS scar, 2 cm	intraperitoneal 300, hemorrhage 4000 erythrocyte 14 U, plasma 1600 ml, cold precipitation 12 U	Heavy vaginal bleeding 2 + h total hysterectomy	10′ 1900 g CPP + PP	67
10	29	15	5	-	CS2, AA2	27	Original CS scar right side, 3 cm	intraperitoneal 3200, hemorrhage 400 erythrocyte 6 U, plasma 800 ml, cold precipitation 12 U	Persistent left lower abdominal pain 1 day, diarrhea 5 times, Vomit once	stillbirth CSP	55
11	32	40^+2^	3	18.4	CS1, AA1	61	Left posterior wall near endocervix, 3 cm, vertical	intraperitoneal 50, hemorrhage 200	—	10′ 3150 g	104
12	29	34^+3^	5	32.5	CS2, AA2	73	Original CS scar, 10 cm	intraperitoneal 1000, hemorrhage 200 plasma 1000 ml	Abdominal pain with vaginal drainage 10 h	stillbirth HELLP syndrome	65
13	33	29^+4^	5	-	VB2, AA2	—	Bottom of the uterus near the right uterine horn, 5 cm	intraperitoneal 1500, hemorrhage 200 erythrocyte 4 U	Persistent lower abdominal pain (slightly to the right around the umbilicus) 5 + h	6’-9’-9′ 1350 g PI, insufficient amniotic fluid	59
14	30	33	7	26.7	CS1 (superimposed preeclampsia), VBAC1, AA3, 2016 Laparoscopic right tubal opening for embryo retrieval	58(VB 28、IC 13)^a^	Right uterine fundus, old, 4 cm, covered by large omental adhesions	hemorrhage 1000	Vaginal bleeding 10 + days	9’-10′ 1990 g CPP	101
15	34	26	6	25.0	VB2, CS1, AA2	46	Original CS scar right side, 1 cm	intraperitoneal 2000, hemorrhage 1000 autologous blood 750 ml, erythrocyte 4 U, plasma 600 ml, cold precipitation 6 U	Abdominal pain 3 h, ultrasound suggests abdominal fluid	5’-9’-10′ 1100 g PP、GDM	69
16	33	35^+5^	7	28.2	CS1 (right uterine horn wedge excision, PPH), AA5	62(IC 36)^a^	Right uterine horn, 3 cm, covered by large omental adhesions	intraperitoneal 50, hemorrhage 300	Bloating 2 days	10′ 2650 g wind heart disease; GDM	117
17	30	40^+3^	4	-	VB2, AA1	—	Posterior wall of the uterus near the ligamentum intrinsicum of the left ovary, 6 cm, vertical	intraperitoneal 500, hemorrhage 1000 erythrocyte 8 U	Premature rupture of membranes after multiple intercourse, oxytocin, changes in fetal position and heart rate	2’-2’-3′ 3600 g	62
18	30	26^+5^	4	-	AA3	—	Left uterine fundus, 1 cm	intraperitoneal 2500, hemorrhage 3000 erythrocyte 12 U, cold precipitation 16 U	abdominal pain with vomiting 7 + h, LMWH	double stillbirth IVF-ET	52
19	31	21	4	-	VB1, AA2	—	Right posterior wall near the cervix, 3 cm	intraperitoneal 500, hemorrhage 1000 erythrocyte 6 U	Bloating 2 days	stillbirth	63
20	31	37	4	-	VB1, AA2	—	Right side of uterus near round ligament, 6 cm	intraperitoneal 1000, hemorrhage 600 erythrocyte 8 U, plasma 600 ml	Breech delivery, difficulty delivering the fetal head	3’-4’-4′ 3550 g	59
21	37	39^+1^	3	38.8	CS1, VBAC1	42(VB 24)^a^	Original CS scar, 2 cm	hemorrhage 200	intrauterine distress	10′ 3700 g	105
22	30	38	5	18.0	CS1, AA3, double uterus (single cervix, double uterine cavity)	30	Connection of the posterior walls of both uterine cavities, 1 cm	hemorrhage 1500 autologous blood 500 ml, erythrocyte 2 U	—	10′ 3450 g CPP, transverse fetal position	82
23	38	23^+3^	7	17.6	CS3 (eclampsia, severe adhesion), AA3	26	Original CS scar near cervix, 3 cm, severe adhesion	intraperitoneal 500, hemorrhage 200	After mifepristone and misoprostol, abdominal pain with vaginal bleeding 7 h, ultrasound shows uterine rupture	stillbirth CHWSP, GDM	97
24	37	32	9	-	VB2, AA5, 2015 Laparoscopic Left Tubectomy	—	Uterine fundus, 12 cm	intraperitoneal 2500, hemorrhage 1500 erythrocyte 8 U, cold precipitation 6 U	Abdominal pain 8 h,vomit once, coma 30 min	stillbirth	76
25	23	34^+1^	5	31.6	VB3, CS1	15	Original CS scar, T-shape, 5 × 3 cm, metal ring	intraperitoneal 500, hemorrhage 500	Lower abdominal pain with vaginal bleeding 1 day, uterine area tenderness	stillbirth	100
26	40	38^+6^	3	18.6	CS1, AA1 (CSP)	156	Left side of original CS scar, 2 cm	intraperitoneal 50, hemorrhage 400	Bloating 2 days	10′ 2850 g adenomyosis, IVF-ET	91
27	32	38^+2^	5	20.5	CS1 (PI), AA3, 2019 transabdominal cerclage	13	Lower part of the posterior uterine wall, 2 cm, left broad ligament, 2 cm	hemorrhage 400	—	10′ 3950 g PA	116
28	33	^31+1^	4	21.7	CS1, AA2	84	Original CS scar, 1 cm	intraperitoneal 500, hemorrhage 200	Ethacridine, uterine area tenderness, ultrasound shows uterine rupture	stillbirth	92
29	36	29	5	19.8	CS2 (PPH), AA2	58	Original CS scar, T-shape, 4 × 5 cm	hemorrhage 800 erythrocyte 6 U	Ethacridine, uterine area tenderness, ultrasound shows uterine rupture	stillbirth	71
30	21	39	4	26.5	CS3, HIV	14	Middle part of anterior wall of uterus, 4 cm	hemorrhage 400	—	10′ 2900 g MPP	80
31	39	37^+2^	4	-	VB2, AA1, 2018UM	—	Lower part of the anterior wall of the uterus near the left broad ligament, 3 cm	intraperitoneal 50, hemorrhage 600	—	10′ 3500 g PPP + P, GDM	93
32	37	21	4	-	CS1, AA2	51	Anterior wall near the left uterine horn to the endocervical os, 10 cm	intraperitoneal 1200, hemorrhage 200 erythrocyte 4 U, cold precipitation 14 U	Spontaneous labor after stillbirth, uterine area tenderness, ultrasound shows uterine rupture	stillbirth CPP	78
33	33	32^+1^	3	19.9	CS2	53	Lower part of the posterior uterine wall, 1 cm	intraperitoneal 3000, hemorrhage 1000 erythrocyte 30 U, plasma 5800 ml, cold precipitation 88 U, platelet 36 U	Tachycardia, bloating, shock litogun, LMWH, total hysterectomy	stillbirth, maternal death, PPP + PP, PGDM	51

Caesarean Section *CS*, Vaginal Birth  *VB*, Artificial Abortion   *AA*, Ectopic Pregnancy   *EP*, Caesarean Scar Pregnancy   *CSP*, Postpartum Hemorrhage  *PPH*, Complete Placenta Previa   *CPP*, Marginal Placenta Previa *MPP*, Pernicious Placenta Previa  *PPP*, Placenta Accreta   PA, Placenta Increta   *PI*, Placenta Percreta  *PP*, Low Molecular Weight Heparin *LMWH*, Hypertensive Disorders of Pregnancy *HDP*, Chronic Hypertension with Superimposed Preeclampsia *CHWSP*, Chronic Hypertension   *CH*, Pregestational Diabetes Mellitus *PGDM*, Gestational Diabetes Mellitus   *GDM*, In Vitro Fertilization and Embryo Transfer   *IVF-ET*, Endometriosis   *EMT*, Uterine Myomectomy   *UM*, Vaginal Birth after Cesarean *VBAC*, Trial Of Labor After Cesarean   *TOLAC*, Induce Childbirth   *IC*, Periconceptional Caesarean Section Scar to Vesicovaginal Fold Distance *CS-VVF*

^a^Case 14: This pregnancy occurred 28 months after vaginal delivery by cesarean section, followed by a induced labor with an interval of 23 months from pregnancy. Case 16: pregnancy was induced 36 months after cesarean section. Case 21: the pregnancy was 24 months after a vaginal delivery following a cesarean section

^b^Fetal outcomes including: Apgar scores and weights of stillbirths and live births

### Causes of uterine rupture

The causes of uterine rupture are shown in Table 3. Ten patients (30.3%) had a history of cesarean section and rupture at an incision site other than the original cesarean section. Patient 2 was involved in a car accident. Patient 11 underwent an elective cesarean section, and uterine rupture was found intraoperatively: the blood flow around the rupture was not rich, the bleeding was not much, and there were no obvious symptoms. Patient 14 had a history of laparoscopic right tubal surgery with poor symptomatology due to adhesion coverage. Patient 16 had a history of cesarean section and wedge resection of the right uterine horn (> 5 years prior), two artificial abortions (AA2), and one induction of labor in middle pregnancy (20^+^ weeks gestation, fetal anomaly, postpartum evacuation), with poor symptomatology due to adhesion coverage. Patient 27 has a cesarean section after transabdominal cerclage (intraoperative discovery of placental implantation), with uterine rupture in the cerclage line. Patient 33 had a history of two cesarean sections; this time, she was treated with ritodrine for fetal preservation and low molecular heparin in an outside hospital due to the presence of contractions, small vaginal bleeding, fast heart rhythm, and incomplete suppression of contractions, which were not taken seriously. She was transferred to our hospital for shock and stillbirth where she underwent an emergency cesarean section.

**Table 3:** Classification of causes of complete uterine rupture in 33 cases

Categorization	Number of cases (rate)	Categorization	Number of cases (rate)
Age (years)		BMI (kg/m^2^)	
< 35	24 (72.73%)	Normal (18.5 ≤ BMI ≤ 23.9)	9 (27.27%)
35 to < 40	7 (21.21%)	Overweight (BMI ≥ 24)	14 (42.42%)
≥ 40	2 (6.06%)	Obese (BMI ≥ 27)	10 (30.3%)
Number of pregnancies		Number of fetuses	
< 3	3 (9.09%)	Singleton	32 (96.97%)
≥ 3	30 (90.91%)	Twins	1 (3.03%)
Number of deliveries		Weeks of pregnancy at the time of uterine rupture (weeks)	
≤ 2	26 (78.79%)	0 ~ 11^+6^	0 (0%)
> 2	7 (21.21%)	12 ~ 27^+6^ /post-partum	7 (21.21%)/2 9.09%)
Number of cesarean sections			
0	8 (24.24%)	28 ~ 36^+6^ /post-partum	15 (45.45%)/3 (9.09%)
1	18 (54.55%)	≥ 37 /post-partum	11 (33.33%)/3 (9.09%)
2	5 (15.15%)	Rupture position	
3	2 (6.06%)	Back wall	6 (18.18%)
Interval from previous CS (years)			
< 1.5	5 (15.15%)	Front wall	15 (45.45%)
1.5 to < 2	0 (0%)	Original CS incision	
2 to ≤ 3	5 (15.15%)	Non-scarred	5 (15.15%)
> 3 and ≤ 5	9 (27.27%)	Uterine fundus	6 (18.18%)
> 5	6 (18.18%)	Many places	1 (3.03%)
Not a history of CS		Current pregnancy	
Open myomectomy	1 (3.03%)	Placenta previa	8 (24.24%)
Laparoscopic tubectomy	1 (3.03%)	Placental/penetrating implantation	8 (24.24%)/4 (12.12%)
Laparoscopic tubal opening and embryo extraction	1 (3.03%)	DM	2 (6.06%)
Laparoscopic cervical cerclage	1 (3.03%)	GDM	3 (9.09%)
Wedge excision of the uterine horn	1 (3.03%)	Placental adhesion	1 (3.03%)
Laparoscopic surgery for endometriosis	1 (3.03%)	Adenomyosis	1 (3.03%)
Other previous medical history		Hysteromyoma	1 (3.03%)
Double uterus (single cervix, double uterine cavity)	2 (6.06%)	IVF-ET	1 (3.03%)
Postpartum hemorrhage and blood transfusion	4 (12.12%)	Use of LMSH	2 (6.06%)
Severe adhesion	3 (9.09%)	Hyperemesis	4 (12.12%)
Placental abnormalities	2 (6.06%)	Twin pregnancy	1 (3.03%)
Caesarean scar pregnancy	1 (3.03%)	Coitus before childbirth	1 (3.03%)
Induction of labor in mid/late pregnancy after CS	2 (6.06%)	Heart disease	1 (3.03%)
VBAC	2 (6.06%)	Caesarean scar pregnancy	1 (3.03%)
Hyperemesis	3 (9.09%)	Traffic accidents	1 (3.03%)
Twin pregnancy	1 (3.03%)	Mental retardation	1 (3.03%)
GDM	1 (3.03%)	Use of oxytocin	1 (3.03%)
History of intrauterine manipulation only	6 (18.18%)	Use of induced abortion drugs	3 (9.09%)
		HIV Transferred to our hospital	1 (3.03%) 13 (39.39%)

Caesarean Section *CS*, Vaginal Birth after Cesarean *VBAC*, Gestational diabetes mellitus *GDM*, diabetes mellitus *DM*, low-molecular heparin sodium *LMSH*

Six patients (18.18%) had a history of uterine operation. Patient 17 had a history of one AA and two vaginal births (VB). She was involved in coitus multiple times in the week prior to the delivery, and the night before delivery, resulting in premature rupture of the membranes; she did not notify the medical staff, and the labor did not come to term. The cervical canal did not open, HS-1 (the lowest point of the fetal skull is 1 cm below the sciatic ischiadica), and 10 min after using oxytocin, cervical dilatation was at 3 cm. Oxytocin was discontinued once abnormal fetal presentation was observed. A cesarean section was performed immediately fetal heart monitoring revealed a deceleration. Patient 20 underwent a breech vaginal trial of labor, with difficulty delivering the fetal head, vaginal rupture, and uterine rupture.

### Clinical signs and symptoms of uterine rupture

The clinical signs and symptoms associated with complete uterine rupture are shown in Table 4.

**Table 4:** Apparent clinical signs and symptoms associated with complete uterine rupture

Symptoms and signs	Number of cases (rate)	Morbidity (reported in the literature)
None	7 (21.21%)	
Abdominal pain/typical persistent lower abdominal pain	12 (36.36%)/4 (12.12%)	58.1 [5] 23% [ 6 ]
Vaginal bleeding	4 (12.12%)	
Shock	4 (12.12%)	
Change in fetal position	1 (3.03%)	
intrauterine distress/Preoperative fetal death	1 (3.03%)/13(39.39%)	23.6 ~ 87% [7]
Ultrasound Signs of Uterine Rupture	4 (12.12%)	36 ~ 77% [8]
uterine area tenderness	4 (12.12%)	36.0% [5]
CS-VVF (after 22 weeks gestation)	26.3 ± 7.1 mm	23.7 ± 3.5 mm [9]

Periconceptional cesearean section scar-to-vesicovaginal fold distance, CS-VVF

There were a few special cases. Patient 1 had an intellectual disability and was unable to express her discomfort accurately. Patient 25 had a metal ring at the breach site. Patient 10 had a cesarean scar pregnancy (CSP) with abdominal blood accumulation (mass) of approximately 3200 ml. Four (12.21%) placental implantation at the incision sites. Three patients presented with severe postpartum hemorrhage, and two of them underwent hysterectomies. The third patient had a repeat vaginal delivery after three VB, two cesarean sections (CS), and one vaginal birth after cesarean (VBAC), and was transferred to our hospital with hemorrhage after delivery; intraoperative rupture of the original cesarean section incision and placenta implantation at the rupture site were observed. Patient 21 had a post-VBAC.

### Treatment of uterine rupture and maternal and fetal outcomes

The fetal outcomes and treatment of uterine rupture are shown in Table 5. Postpartum hemorrhage did not occur in 11 patients (33.33%); six (18.18%) were found to have uterine rupture during full-term, elective surgery, with little blood flow around the rupture, and little bleeding with no obvious symptoms. Two patients (6.06%) had severe adhesions. One (Patient 21) was promptly delivered by cesarean section due to abnormal fetal heart rate monitoring; and two (Patients 28 and 29) had uterine tenderness after ethacridine administration and promptly underwent cesarean section.

**Table 5:** Treatment and maternal and fetal outcomes

Categorization	Number of cases (rate)
No transfusion	12 (36.36%)
Transfusion	21 (63.64%) (history of CS 14 cases)
Massive blood transfusion	10 (30.3%) (documentation 61.8% ~ 92.5% [5, 10])
Transfusion components	
erythrocytes	19 (57.58%)
plasma	10 (30.3%)
platelet	1 (3.03%)
cold precipitation	7 (21.21%)
autologous blood	2 (6.06%)
Bleeding volume < 1000 ml	11 (33.33%)
≥ 1000 ml	22 (66.67%)(documentation 43.3 [11])
1000 ~ 2000 ml	11 (33.33%)
> 2000 ml	11 (33.33%)
Hysterectomy (particular year)	7 (21.21%) (documentation 9.5% ~ 21.2% [2, 5])
2015	5 (15.15%)
2016	1 (3.03%)
2017 ~ 2021	0
2022	1 (3.03%)
Total hysterectomy	5 (15.15%)
Subtotal hysterectomy	2 (6.06%)
Preoperative fetal death	13 (39.39%)
Gestation week ≥ 28 weeks	8 (24.24%)
Spontaneous contractions after fetal death	2 (6.06%)
Perinatal deaths	15 (45.45%) (documentation 26.2% ~ 83.6% [12, 13])
Fetal malformation induced labor	3 (9.09%)
Neonatal severe asphyxia	2 (6.06%, full-term gestation) (documentation 25.2 [5])
Maternal death	1 (3.03%) (documentation 1.2% ~ 15.9% [2, 14])

## Discussion

### Incidence of complete uterine rupture

Since the opening of the separate two-child policy in 2013, full liberalization of the two-child policy in 2016, and opening of the three-child policy in 2021, the cesarean section rate in China has increased from 34.9% (2014) to 41.1% (2016) [15]. Following this, the rate of scarred uterus has increased from 9.8% (2012) to 17.7% (2016) [16], which is far beyond the World Heallth Organizations ideal range.

The incidence of uterine rupture has been reported in several countries and regions; it is not consistent across countries and regions. This rate is related not only to the high-risk factors of the pregnant women themselves, but also to the economic level of each country, number of years of occurrence (which is related to the country's policy at that time), level of medical care, and transportation status (referral time). The reported incidence of uterine rupture: is 0.06% in Northern Europe [17], 0.67% in the University of Pakistan Teaching Hospital [18], 0.01% in the First Maternity and Infant Hospital of Shanghai [19], 0.2% in the Jiangxi Maternity and Child Healthcare Hospital [20], and 0.05% in the Women's Hospital of the Medical College of Zhejiang University [21]. Following the latest data [22], the total incidence of uterine rupture in China is 0.13%, consistent with 0.1% found in this study.

### Analysis of the etiology and risk factors for complete uterine rupture

Due to the low cesarean section rate and the large number of patients with two or multiple deliveries between 1960 and 1990, uterine rupture was the predominantly primary. After 1990, with the implementation of family planning policies, the cesarean section rate increased, and subsequently, cesarean section scar rupture became the primary cause of uterine rupture [23]. Therefore, with the improvement in medical standards, doctors' awareness of uterine rupture, and repeated emergency drills, the main etiology has changed.

The known etiologies and risk factors for uterine rupture are as follows [1, 23].

1. *Previous uterine injury or history of abnormalities.*

A history of myometrial surgery and short or long intervals between surgeries, which include cesarean section (incidence of uterine rupture was 0.071% [21], 0.095% for a history of one cesarean Sect [24]., and 1.92% for a history of two or more cesarean Sects [24].), history of repair of uterine rupture (33% [25]), myomectomy, wedge resection of the uterine horn (incidence of uterine rupture is up to 30% [26]), tubal surgery, hysteroscopic septum resection (incidence of uterine rupture is 1.0%–2.7% [27, 28]), and separation of adhesions in the uterine cavity. Human Immunodeficiency Virus (HIV) infection may increase the incidence of cesarean delivery complications [29]. Current research suggests that the uterine wound healing takes 12 weeks [30], while myometrial incision healing and scar formation takes 6–12 months [31]; however, wound healing does not mean that it is able to withstand the pressure of pregnancy immediately. It also undergoes a process of tissue reconstruction, which further strengthens the elasticity of the uterine myometrial wall in the area of the scar. Therefore, less than 12–18 months is a high-risk factor for uterine rupture [32], and 2–3 years after cesarean section is the optimal period for uterine incision healing [33]. After > 5 years [6], the uterine scar’s degree of muscularization will gradually deteriorate and it will lose elasticity, making uterine rupture more likely during another pregnancy. The risk of uterine rupture for second pregnancies has been reported in the literature, even when tubal surgery does not involve the mesosalpinx or uterine horn, with a higher risk in the presence of electrocoagulation injuries, injury to or absence of part of the myometrial layer of the uterine horn, localized unsutures, and short intervals between pregnancies. The incidence of uterine rupture in our study was 0.79 per 1000 in those with a history of cesarean section, 0.057% in those with a history of one cesarean section, and 0.022% in those with a history of two or more cesarean sections. In our study, 11 patients (33.33%) had an interval of pregnancy out of 1.5 and 5 years, two (6.06%) had a history of tubal surgery, one (3.03%) had a history of wedge resection of the uterine horn, and one (3.03%) was a person living with HIV. It is important to note that in patients with a history of uterine rupture, the rupture was not at the same location. Uterine injuries include abortion, curettage, as well as sharp or blunt contusions such as car accidents, knives, and hidden uterine ruptures. In the present study, six (18.18%) patients had a history of intrauterine manipulation as a risk factor. Congenital abnormalities include uterine dysplasia, and connective tissue defects. In the present study, two patients (6.06%) had a double uterus. Furthermore, one patient (case 7) had a history of transabdominal cervical cerclage. During pregnancy, the cerclage line increases with the uterus, creating a chronic transverse cutting effect on the cervix, Uterine rupture occurs at the site of the cervical cut once there is significant uterine contraction.

(2) *Combined uterine injuries or abnormalities in this pregnancy*

- Postnatal etiologies include advanced age, muliple pregnancies and deliveries, spontaneous tonic uterine contractions, excessive contractions due to the use of oxytocin or prostaglandins and maternal sensitivity to drugs, prostaglandin or saline intra-amniotic infusion, sharp forceps contusion, external inversion, amniotic fluid overload, or multiple pregnancies. In this study, one patient (3.03%) was treated with uterotonics and three (9.09%) underwent induction of labor.
- Intrapartum etiologies include any mechanism leading to obstruction of fetal descent, such as pelvic stenosis, cephalopelvic disproportion, obstruction of the soft birth canal, abnormal fetal position, and macrosomia; internal inversion; forceps delivery; emergency labor; breech traction; fetus destruction; excessive dilatation of the uterus in the lower part of the uterus caused by fetal anomalies; excessive pressure in the uterine cavity during delivery; implantation of the placenta or severe adhesion; and difficulty in manually stripping the placenta. One patient (case 20) in this study had uterine rupture due to breech traction during vaginal delivery and eight (24.24%) had placenta implantation.
- Acquired etiologies include gestational trophoblastic disease, adenomyosis, posterior flexion uterine implantation, and uterine artery embolization surgery. One patient in this study had adenomyosis.
- CSP involves a poorly healed uterine incision, wide scarring, and inflammation, leading to the development of microscopic fissures through which the fertilized ovum is deposited into the myometrium. Most cases have a poor prognosis [1]. Only one case of CSP was reported in this study.
- Placental implantation: when it occurs at the site of the original cesarean section scar is caused by a structural defect in the endometrium [23] that allows the placenta to attach abnormally to the uterine myometrium. In our study, four patients (12.12%) had placental implantation, with three who had severe postpartum hemorrhage and two who underwent hysterectomies.
- Trial of labor after cesarean (TOLAC), during a second pregnancy or request for a vaginal trial of labor; A meta-analysis showed that TOLAC results in a 0.27% higher risk of uterine rupture [9]. The management of TOLAC is a multifactorial. Factors [34, 35] such as a previous vaginal delivery, use of epidural anesthesia, indication for previous cesarean section, pregnancy complications (such as preeclampsia, and placental anomalies), fetal weight above 4000 g, dose of oxytocin used, induction of labor with prostaglandins, women who delivered at > 41 ^+0^ weeks of gestation, ethnicity, cervical length, head-perineum distance, maternal age (maybe), inter-delivery interval, body mass index (maybe), and prolonged second stage of labor (maybe) contribute to uterine rupture during TOLAC. However, there are no data or literature supporting whether to perform a TOLAC and assess the risk of uterine rupture in a second pregnancy after a history of two cesarean deliveries and after one VBAC.

(3) Others

Endometriosis causes tissue adhesions. Surgical separation of these adhesions results in localized myometrial destruction and thinning, affecting the healing, brittleness, and elasticity of the scar. Patient 6 had this clinical presentation. It has also been shown that the distance from the cesarean scar to the vesicovaginal fold (suggestive of the horizontal position of the uterine incision from the previous cesarean section) is significantly increased in patients with a gestational age > 22 weeks and antepartum uterine rupture, and may be predictive of uterine rupture [9].

Regardless of how the uterus is damaged, scarring occurs during the repair process, which constitutes non-normal muscle tissue (connective and scar tissue) [1]. This forms a weak site of the uterus during pregnancy. We obseerved that uterine rupture occurred at a random site, mainly at the weakest point of the uterus. There is no effective means for detecting or predicting the weakest point of the uterus. In addition, the uterus may have ruptured in more than one location (Patient 2 had three ruptures).

### Clinical manifestations and early diagnosis of uterine rupture

In general, most uterine ruptures progress from uterine rupture precursors, with the main clinical manifestations being abdominal pain (especially during the intervals between contractions), uterine tenderness (reported in the literature to be approximately 36.0% [21]), fetal abnormalities, abnormal vaginal bleeding, pathological contractions, hematuria, disappearance of contractions, hemodynamic instability (tachycardia, hypotension, or shock), change of fetal position, signs of uterine rupture detected by ultrasound, and changes in abdominal contour [1]. Some symptoms are asymptomatic; however, typical symptoms are rare (less than 10% [36]). Currently, pregnancy relies on the co-monitoring of history, clinical presentation, signs, and ultrasonography or magnetic resonance imaging (MRI). It is very difficult to rely on pregnancy management to prevent uterine rupture, which may be due to the following: the timing of uterine rupture is random, the rupture may be unrelated to the original surgical site, most patients have multiple risk factors, and the weakest part of the uterus may change with gestational age and cannot be predicted in advance. The time window for uterine rupture is difficult to control. The time may be longer in patients with thick abdominal fat and varying pain tolerance. If referral is required (long travel time), the optimal time for resuscitation is easily delayed. Abdominal pain, fetal distress, and vaginal bleeding do not allow us to initially consider uncommon uterine ruptures. Clinicians have limitations in considering abdominal pain, which is prone to misdiagnosis as other acute abdominal conditions or labor precursors. Furthermore, multiple pregnancies, literacy levels, and family economic status may lead to irregularities during obstetrical tests. Uterine rupture most often occurs suddenly most ofter, with immediate surgery performed upon diagnosis, failure to achieve continuous fetal heart monitoring, or fetal death at the time of presentation. Other conditions that do not directly lead to uterine rupture but can interfere with early recognition, such as mental retardation, history of frequent coitus, history of abdominal trauma, unawareness of the condition by family members (inability to provide an accurate history when the patient is in shock), use of ritodrine (rapid heart rate) and vomiting during pregnancy, can mask the early signs of shock. Color ultrasonography and MRI can be affected by the level of the examiner, thickness of abdominal fat in the pregnant woman, measurement site, number of measurements, bladder filling, rupture site (posterior wall rupture is difficult to diagnose), fetal movement, dynamic monitoring or not, and clarity of the ultrasound machine. In addition, the presence of unknown previous surgery (inverted T-shaped incision, uterine monolayer suture, weak local myometrium, infection, poor healing of incision, cause of postpartum hemorrhage, and method used to stop bleeding), diverticulum of the uterine incision (occurs in approximately 60% of patients after a primary cesarean section and 100% after three cesarean Sects [37].), any perforation during uterine manipulation, artificial placental removal during previous delivery, and subsequent follow-ups can affect the diagnosis of uterine rupture. In particular, the healing of the original cesarean section scar is unknown; a study showed that the use of synthetic absorbable monofilament sutures for uterine closure was associated with increased residual myometrial thickness, with respect to synthetic absorbable multifilament sutures. A uterine segment thickness after cesarean section below 2.0 mm between 35 and 38 gestational weeks has been repetitively associated with a greater risk of uterine rupture or scar dehiscence [37]. Furthermore, when the breach is small and there are no blood vessels at the breach, there may be no obvious symptoms or imaging changes. Atypical symptoms, difficult diagnosis of intra-abdominal hemorrhage, and unsupportive ancillary tests plague surgical decision-making. There is limited data on some factors that may affect the healing of the uterine incision [23] (previous history of postpartum hemorrhage, gestational diabetes mellitus or diabeties as a comorbidity, embryo transplantation, hypertensive disorders of pregnancy, and hypoproteinemia), and factors that may provide local protection, such as the severity of the pelvic-abdominal adhesions (three patients in this study had little hematochezia or peritoneal hemorrhage). Therefore, the education of pregnant women and their families, as well as the rapid recognition of uterine rupture after it occurs, are key to early diagnosis. 

### Complications of complete uterine rupture

Uterine rupture can cause severe postpartum hemorrhage, shock, disseminated intravascular coagulation, impaired organ function (ischemia–reperfusion), bladder injury, massive blood transfusion, hysterectomy, maternal death, neonatal asphyxia, ischemic-hypoxic encephalopathy, perinatal death (fetal or neonatal death), and other serious adverse outcomes.

## Conclusions

Good prenatal and pregnancy care (contraceptive promotion, previous surgical records, control of diet weight gain, etc.), graded management (all women need to be risk-graded, strict control of high-risk factors, and timely referral), and early hospitalization of patients with high-risk factors for uterine rupture are key to the early diagnosis and treatment of uterine rupture. For patients with reproductive requirements, strict control of surgical indications, strengthening of suturing skills, guidance on postoperative precautions, strict control of indications for uterotonics and close monitoring are important. Correct management of the labor process, mastery of the indications for obstetric surgically assisted delivery and operation norms, and strict inspection during surgery (e.g., abdominal cervical cerclage patients to check the integrity of the lower segment of the uterus in the posterior wall) are also required. Regardless of high-risk factors, vigilance for uterine rupture, early recognition, proactive management, and training of rapid response teams should be strengthened to achieve favorable maternal and fetal outcomes.

## Data Availability

All data generated or analysed during this study are included in this published article.

## Abbreviations

AA: Artificial Abortion
CS: Caesarean Section
CSP: Caesarean Scar Pregnancy
CPP: Complete Placenta Previa
CS-VVF: Caesarean Scar to Vesicovaginal Fold Distance
CH: Chronic Hypertension
CHWSP: Chronic Hypertension with Superimposed Preeclampsia
EP: Ectopic Pregnancy
EMT: Endometriosis
GDM: Gestational Diabetes Mellitus
PGDM: Pregestational Diabetes Mellitus
HDP: Hypertensive Disorders of Pregnancy
IC: Induce Childbirth
IVF-ET: In Vitro Fertilization and Embryo Transfer
LMWH: Low Molecular Weight Heparin
MPP: Marginal Placenta Previa
PA: Placenta Accreta
PI: Placenta Increta
PPP: Pernicious Placenta Previa
PPH: Postpartum Hemorrhage
TOLAC: Trial Of Labor After Cesarean
UM: Uterine Myomectomy
VB: Vaginal Birth
VBAC: Vaginal Birth after Cesarean

## References

[1] Xie Xing, Kong Beihua, Duan Tao. Obstetrics and gynecology. 9th ed. Beijing: people’s health press, 2018:212–213

[2] Zhe L, Huixia Y, Hong X (2019). National multicenter survey on the status of uterine rupture and analysis of outcomes. Chinese Journal of Obstetrics and Gynecology.

[3] Andrea Tinelli, Loannis P. Kosmas, Jose Tony Carugno, et at. Uterine rupture during pregnancy: The URIDA (uterine rupture international data acquisition) study[J]. Int J Gynecol Obstet 2021,1(157):76–84, DOI:10.1002/IJGO.13810 10.1002/ijgo.13810 34197642

[4] Jian-Ping Z, Juhua W (2011). Diagnosis and treatment of uterine rupture. Chinese Journal of Practical Gynecology and Obstetrics.

[5] Tiantian C, Chunrong L, Yijun L, Meng C, Xinghui L (2020). Analysis of clinical characteristics of 86 cases of complete uterine rupture[J]. Journal of Practical Obstetrics and Gynecology.

[6] Perdue M, Felder L, Berghella V (2022). First-trimester uterine rupture: a case report and systematic review of the literature [J]. Am J Obstet Gynecol.

[7] Savukyne  E, Bykovaite·stankeviciene R, Machtejeviene E (2020). Symptomatic uterine rupture: a fifteen year review [J]. Medicina (Kaunas).

[8] Sharon N, Maymon R, Pekar-Zlotin M (2022). Midgestational prelabor spontaneous uterine rupture: a systematic review [J]. J Matern Fetal Neonatal Med..

[9] Vimercati A, Dellino M, Crupano FM, Gargano G, Cicinelli E (2021). Ultrasonic Assessment Of Cesarean Section Scar To Vesicovaginal Fold Distance: An Instrument To Estimate Pre-Labor Uterine Rupture Risk. J Matern Fetal Neonatal Medicine.

[10] Singh A, Shrivastava C (2015). Uterine rupture: still a harsh reality![J]. J Obstet Gynaecol India.

[11] Al-Zirqi I, Daltveit AK, Vangen S (2019). Maternal outcome after complete uterine rupture[J]. Acta Obstet Gynecol Scand.

[12] Al-Zirqi I, Daltveit  AK, Vangen S (2018). Infant outcome after complete uterine rupture[J]. Am J Obstet Gynecol.

[13] Xinghui L, Jing He, Hongbo Qi (2018). Midwifery.

[14] Li HT, Luo S, Trasande L (2017). Geographic Variations and Temporal Trends in Cesarean Delivery Rates in China, 2008–2014. JAMA.

[15] Liang J, Mu Y, Li X, et al. Relaxation of the one child policy and trends in caesarean section rates and birth outcomes in China between 2012 and 2016: Observational study of nearly seven million health facility births.BMJ,2018,360(5):k817 10.1136/bmj.k817 PMC5836714 29506980

[16] Colmorn LB, Petersen KB, Jakobsson M (2015). The Nordic Obstetric Surveillance Study: a study of complete uterine rupture, abnormally invasive placenta, peripartum hysterectomy, and severe blood loss at delivery. Acta Obstet Gynecol Scand.

[17] Aziz N, Yousfani S (2015). Analysis of uterine rupture at university teaching hospital Pakistan. Pak J Med Sci.

[18] Qingling K, Yang Z, Lei F (2019). Analysis of complete uterine rupture in middle and late pregnancy. Progress of modern obstetrics and gynecology.

[19] Meilong Xu (2018). Clinical analysis of 82 cases of uterine rupture in pregnancy. China Maternal and Child Health.

[20] Xiaoxia B, Zhengping W, Xiaofu Y (2014). Clinical analysis of 67 cases of uterine rupture. Chinese Journal of Obstetrics and Gynecology.

[21] Zhou Y, Mu Y, Chen P (2021). The incidence, risk factors and maternal and foetal outcomes of uterine rupture during different birth policy periods: an observational study in China. BMC Pregnancy Childbirth.

[22] Huixia Y, Hongbo C, Qintian Z (2020). Williams Obstetrics [M].

[23] Suqin Wu, Ying W, Zhiming S (2017). Risk factor analysis of mode of delivery for second pregnancy in scarred uterus. Chinese Journal of Clinical Pharmacology.

[24] Usta IM, Hamdi MA, Musa AA (2007). Pregnancy outcome in patients with previous uterine rupture[J]. Acta Obstet Gynecol Scand.

[25] Liao CY, Tse J, Sung SY (2017). Cornual wedge resection for interstitial pregnancy and postoperative outcome[J]. Aust N Z JObstet Gynaecol.

[26] Propst AM, Liberman RF, Harlow BL (2000). Complications of hysteroscopic surgery: predicting patients at risk[J]. Obstet Gynecol.

[27] Jansen FW, Vredevoogd CB, van Ulzen K (2000). Complications of hysteroscopy: a prospective, multicenter study[J]. Obstet Gynecol.

[28] A Vimercati, P Greco, G Loverro, Pl Lopalco, V Pansini, L Selvaggi. Maternal Complications After Caesarean Section In Hiv-Infected Women. Europ J Obstet Gynecol Repr Biol (90) 2000:73–76 10.1016/s0301-2115(99)00229-8 10767514

[29] Tsuji S, Takahashi K, Imaoka I (2006). MRI evaluation of the uterine structure after myomectomy[J]. Gynecol Obstet Invest.

[30] Chaoxia L, Danqing C (2018). Timing and mode of delivery after uterine trauma. Journal of Practical Obstetrics and Gynecology.

[31] Cunningham S, Algeo CE, Defranco EA (2021). Influence of inter pregnancy interval on uterine rupture[J]. J Matern Fetal Neonatal Med.

[32] Hasbargen U, Margarita SM, Peter H (2002). Uterine dehiscence in a nullipara, diagnosed by MRI, following use of unipolar electrocautery during laparoscopic myomectomy[J]. Hum Reprod.

[33] Kui-lin F, WeiShe Z (2017). Repregnancy in elderly women with scarred uterus. Chinese Journal of Practical Gynecology and Obstetrics.

[34] Gitas G, Alkatout I, Ertan KA (2022). Risk factor analysis in women who underwent trial of labor after cesarean section: a multicenter study in Germany[J]. J Turk Ger Gynecol Assoc.

[35] Gitas G, Proppe L, Ertan AK (2021). Influence of the second stage of labor on maternal and neonatal outcomes in vaginal births after caesarean section: a multicenter study in Germany[J]. BMC Pregnancy Childbirth.

[36] American College of Obstetricians and Gynecologists. ACOG Practice bulletin no. 115: Vaginal birth after previous cesarean delivery[J]. Obstet Gynecol,2010,116(2Ptl):450–463 10.1097/AOG.0b013e3181eeb251 20664418

[37] Antonio Simone Laganà, Antonella Cromi, Roberto Tozzi, et al. Uterine scar healing after cesarean section: managing an old surgery in an evidence-based Environment[J]. Journal of Investigative Surgery,2019,8(32):770–772, 10.1080/08941939.2018.1465145 10.1080/08941939.2018.1465145 29741973

